# The Thermoelectric Properties of Monolayer MAs_2_ (M = Ni, Pd and Pt) from First-Principles Calculations

**DOI:** 10.3390/nano10102043

**Published:** 2020-10-16

**Authors:** Qiang-Lin Wei, Heng-Yu Yang, Yi-Yuan Wu, Yi-Bao Liu, Yu-Hong Li

**Affiliations:** 1 School of Nuclear Science and Technology, Lanzhou University, Lanzhou 730000, China; weiql15@lzu.edu.cn; 2 Engineering Research Center of Nuclear Technology Application, Ministry of Education, East China University of Technology, Nanchang 330013, China; ybliu@ecut.edu.cn; 3 School of Materials Science and Engineering, Hunan University of Science and Technology, Xiangtan 411201, China; yhyyangyu@gmail.com

**Keywords:** the thermoelectric property, thermal conductivity, thermoelectric figure of merit

## Abstract

The thermoelectric property of the monolayer MAs_2_ (M = Ni, Pd and Pt) is predicted based on first principles calculations, while combining with the Boltzmann transport theory to confirm the influence of phonon and electricity transport property on the thermoelectric performance. More specifically, on the basis of stable geometry structure, the lower lattice thermal conductivity of the monolayer NiAs_2_, PdAs_2_ and PtAs_2_ is obtained corresponding to 5.9, 2.9 and 3.6 W/mK. Furthermore, the results indicate that the monolayer MAs_2_ have moderate direct bang-gap, in which the monolayer PdAs_2_ can reach 0.8 eV. The Seebeck coefficient, power factor and thermoelectric figure of merit (ZT) were calculated at 300, 500 and 700 K by performing the Boltzmann transport equation and the relaxation time approximation. Among them, we can affirm that the monolayer PdAs_2_ possesses the maximum ZT of about 2.1, which is derived from a very large power factor of 3.9 × 10^11^ W/K^2^ms and lower thermal conductivity of 1.4 W/mK at 700 K. The monolayer MAs_2_ can be a promising candidate for application at thermoelectric materials.

## 1. Introduction

The energy crisis has always been a problem that plagues the world, so countries around the world are committed to developing new and advanced renewable energy conversion technologies. As an effective energy conversion technology, thermoelectric (TE) power generation has attracted extensive attention from researchers [1,2]. The conversion efficiency between heat and electricity is usually determined by the thermoelectric figure of merit (ZT),

ZT = S^2^σT/(κ_l_ + κ_e_) (1)

ZT depends on the Seebeck coefficient (S), absolute temperature (T), electrical conductivity (σ), thermal conductivity (κ_l_), and electrical conductivity (κ_e_) [3,4]. Generally, a good TE material should have both low thermal conductivity and high Seebeck coefficient. However, the existence of strong coupling effect between each transport parameter within TE materials makes it is not feasible for increasing the ZT value by lifting a certain transport parameter. Several commonly used methods to improve ZT are mainly to optimize the electrical transport performance through belt structure engineering [5,6], and/or to suppress the thermal conductivity of materials through low-dimensional technology [7,8].

It is well known that the successful peeling of graphene greatly promotes the exploration of two-dimensional (2D) layered materials. The special physical, chemical, electronic and optical properties exhibited by the two-dimensional materials make it have good application prospects in the fields of electrodes [9,10], nano devices [11] and TE technology [12]. Hicks and Dresselhaus proposed that ZT value would be improved if 2D structural materials were used instead of three-dimensional ones [13,14]. Because the restriction of carriers in the low-dimensional quantum well leads to the change of energy state density distribution, under certain conditions of Fermi energy, it is beneficial to increase the number of carriers and improve the conductivity and ZT value [15]. At present, the theoretical prediction of new two-dimensional thermoelectric materials with attractive properties such as narrow a band gap and high fluidity is still a very active research field. For example, 2D structures such as silicones [16,17], phosphorene [18,19] and group-IV monochalcogenides (MX, M = Ge, Sn; X = S, Se) [20,21] have a suitable band gap, which effectively promotes them in the field of TE materials applications.

Discovering the application potential of new two-dimensional structural materials in various fields from experiments and theories has always been a research hotspot. The 2D materials currently reported are mostly square or hexagonal lattices. However, the recently discovered pentagonal 2D materials [22,23,24,25] exhibit some interesting properties and have attracted widespread attention. Qian et al. [26] reported theoretically that the 2D binary MX_2_ (M = Ni, Pd, Pt; X = P and As) exhibited a beautiful pentagonal ring network with a narrow direct band gap of about 0.3–0.8 eV and ultra-high mobility for holes and electrons. Based on these intrinsic features, they may have good TE performance. In this paper, we systematically study the TE properties of the monolayer MAs_2_ (M = Ni, Pd, Pt) using the first-principles and Boltzmann transport methods. We find that single-layer NiAs_2_, PdAs_2_ and PtAs_2_ have extremely low lattice thermal conductivities, which are 5.9 W/mK, 2.9 W/mK and 3.6 W/mK, respectively. Meanwhile, the single-layer PdAs_2_ exhibits excellent n-type thermoelectric material characteristics, and the ZT value can reach 2.1 at 700 K. Our research results provide a strong theoretical basis for the experimental exploration of the thermoelectric properties of 2D MAs_2_, and help to promote further experimental verification.

## 2. Computational Methods

The first principles calculations of the monolayer MAs_2_ (M = Ni, Pd, Pt) are presented by density functional theory (DFT) as performed in the Vienna Ab-initio Simulation Package (VASP 5.4.4, Hafner Group at the University of Vienna, Vienna, Austria) [27]. The projector augmented wave (PAW) pseudopotentials are employed to represent the ion-electron interaction [28,29]. The generalized gradient approximation of Perdew-Burke-Ernzerhof (PBE) [30] to the exchange-correlation functional are used. In addition, the cutoff energy and the convergence criteria are set to 500 and 10^−5^ eV, respectively. To obtain a more accurate band gap, the Heyd-Scuseria-Ernzerhof (HSE06) [31] screened hybrid functional was employed. The electricity transport properties are described by Boltzmann transport theory and the relaxation time approximation within the BoltzTraP code [32]. A dense *k*-mesh of 35 × 35 × 1 is chosen to carry out the calculation.

The Boltzmann transport equation is employed for calculating the lattice thermal conductivity, and we use the harmonic second-order interaction force constants (2nd IFCs) and the anharmonic third-order IFCs (3rd IFCs) to be input as performed in ShengBTE code [33] with a dense 50 × 50 × 1 *k*-mesh. Among them, the 3 × 3 × 1 supercell and 3 × 3 × 1 *k*-mesh are adopted to calculate the 2nd IFCs as implemented in PHONOPY code [34], and the thirderdor.py is used to compute the 3rd IFCs with the 3 × 3 × 1 supercell, while the 6th nearest neighbors are considered. The effective masses are derived from the band structure [35]. The carrier mobility is calculated by the deformation potential (DP) theory [36]. The constant scattering time approximation is used to calculate the constant relaxation time [37].

## 3. Results and Discussions

### 3.1. Crystal and Electronic Structures

**Figure 1:**
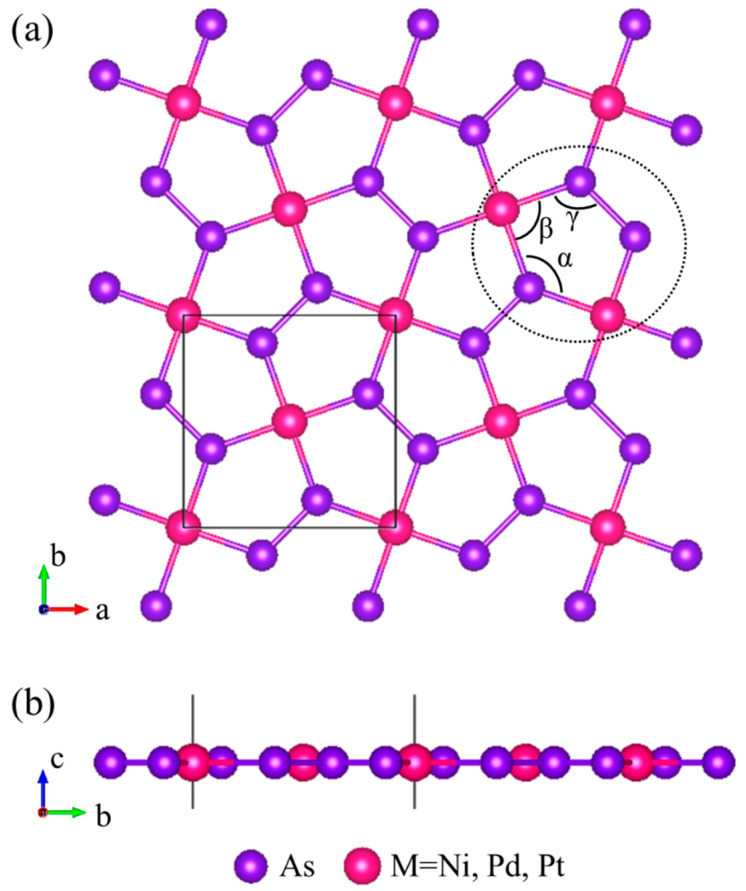
(**a**) Top and (**b**) side views of the monolayer MAs_2_ (M = Ni, Pd, Pt). The pentagonal unit is marked by a black ellipse.

The new 2D monolayer materials MAs_2_ (M = Ni, Pd, Pt), which was obtained by mechanical stripping method, that exhibited good kinetic and thermal stability [26]. The optimal geometry structures of the monolayer MAs_2_ (M = Ni, Pd, Pt) are obtained as showed in Figure 1. From the top view as shown in Figure 1a, it is clear that the monolayer MAs_2_ has a tetragonal structure (space group *Pa3No.*) and each M atom adopts a planar tetra-ligand with four As atoms. A unit-cell is constituted by two M and four As atoms, while all M atoms are always in one plane with As atoms, as shown in Figure 1b. Three As atoms and two M atoms form a pentagonal ring network, marked with a black ellipse, as shown in Figure 1a. The optimized lattice structure parameters are shown in Table 1. The calculation results of the lattice constant (LC) of MAs_2_ and the degrees of the α, β and γ angles of the pentagonal unit are consistent with the calculation results of Qian et al. [26].

**Figure 2:**
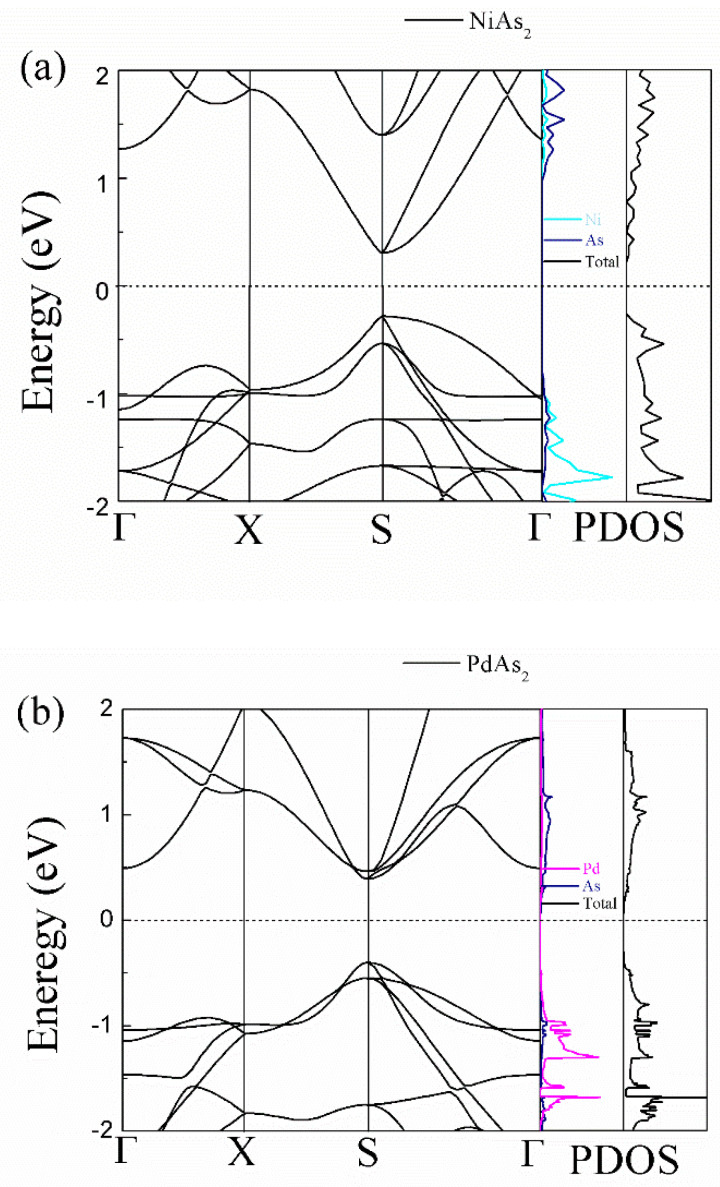

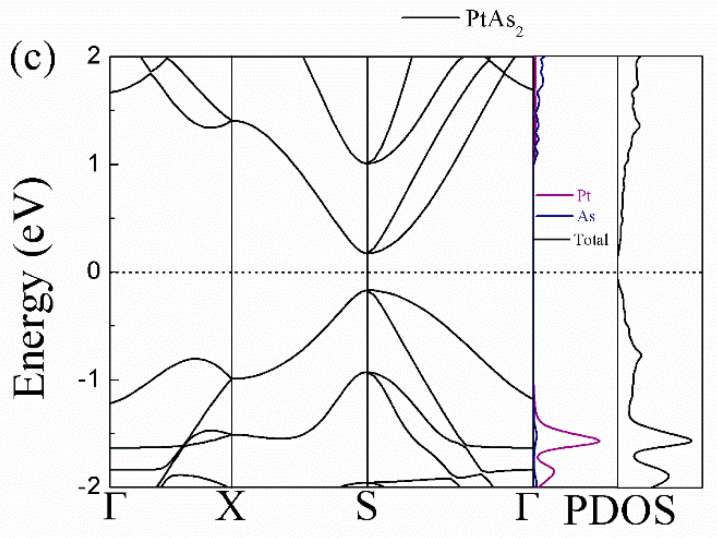
Electronic band structures and projected density of states (PDOS) calculated by HSE06 hybrid functional potentials of monolayer (**a**) NiAs_2_, (**b**) PdAs_2_ and (**c**) PtAs_2_. The zero of the energy in the figures is chosen as the middle of the calculated band gap.

The band structure and projected density of states (PDOS) are computed by using HSE06 as plotted in Figure 2. The presence of direct bang-gap at the S point is clearly visible, which signifies that the monolayer MAs_2_ is the semiconductor, and the corresponding band gaps are 0.59, 0.80 and 0.34 eV for the monolayer NiAs_2_, PdAs_2_ and PtAs_2_, respectively. The band gaps of MAs_2_ materials is within the ideal band gap range for good thermoelectric materials (0.3–1.0 eV) [6]. Among them, the result of PdAs_2_ is very consistent with band gap 0.80 eV by Yuan et al. [38] and 0.78 eV by Pan et al. [39]. It can be seen that the monolayer MAs_2_ possess the great band degeneracy that appears in the valence band (VB) along the X-S direction and primarily originates from the M-orbitals. This kind of band degeneracy means that is MAs_2_ characterized by excellent thermoelectric performance. At present, the band degeneracy has been enhanced by band engineering with the aim of increasing the flatness of the density of states (DOS) to improve the power factor (PF). From the PDOS, a high density of states near the Fermi level are mainly contributed by the M-orbitals, whereas the As have only a small contribution. Besides, there are spikes near the Fermi energy level that can be observed, which effectively promotes the sharp increasing of Seebeck coefficient.

### 3.2. Electrical Transport Properties

The electronic properties can be characterized on the basis of carrier mobility for monolayer MAs_2_, along the conveyor directions. We calculate them using the deformation potential (DP) theory proposed by Bardeen and Shockley [36]. The formula of carrier mobility in 2D systems can be written as follows [35,40]:

(2) $$\mu_{2D}=\frac{e\hbar^{3}C^{2D}}{\kappa_{B}Tm^{*}m_{d}E_{l}^{2}}$$

where *k*_B_ is the Boltzmann constant, T represents the temperature that is taken as 300 K, m* is the effective mass for the conveyor direction, *m_d_* is the average effective mass defined by $m_{d}=\sqrt{m_{x}m_{y}}$, *E_l_* is the deformation potential constant, and *C*^2*D*^ is the effective 2D elastic constants, respectively. The calculated effective mass, carrier mobility and relaxation time (τ = μm*/e) are shown in Table 2. After calculation, it was concluded that the electrical transport properties of MAs_2_ are isotropic, which results from its perfect lattice symmetry, m_d_ is equal to m*. Noticeably, it shows a high hole mobility (34.27 cm^2^/Vs) of PdAs_2_ at room temperature, which is much higher than that of NiAs_2_ (~1.93 cm^2^/Vs) and PtAs_2_ (~4.80 cm^2^/Vs). The high mobility in monolayer PdAs_2_ is associated with the ideal band gap, which is beneficial to its electrical transport, while the mobilities of the holes are PtAs_2_ and showed a high hole mobility (~17.07 cm^2^/Vs) at room temperature, which is much higher than that of NiAs_2_ (~1.93 cm^2^/Vs) and PtAs_2_ (~4.80 cm^2^/Vs).

**Figure 3:**
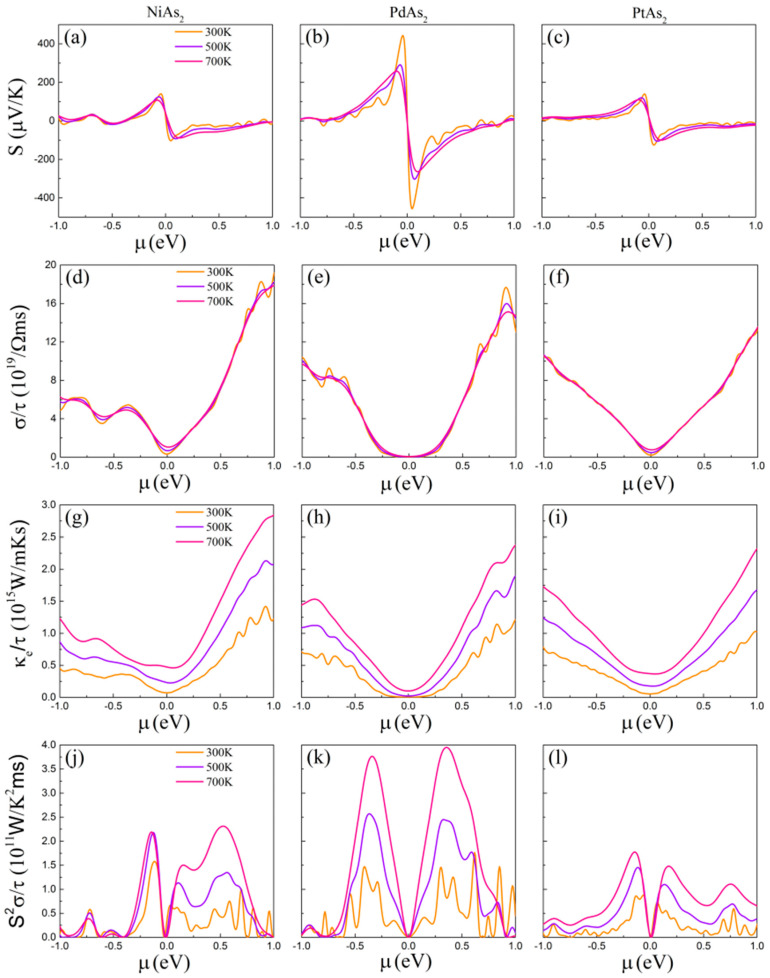
(**a**–**c**) Seebeck coefficients, (**d**–**f**) electrical conductivity, (**g**–**i**) electronic thermal conductivity, and (**j**–**l**) power factor with respect to the scattering time as functions of the chemical potential µ of monolayer MAs_2_.

Based on the Boltzmann transport equation and rigid band approximation, the electricity transport properties under the relaxation time approximation are calculated. After calculation, it was found that the electrical transport properties of MAs_2_ are isotropic, which results from their perfect lattice symmetry. As shown in Figure 3, we can find that the Seebeck coefficient (S), electrical conductively (σ/τ), electron thermal conductively (κ_e_/τ) and power factor (S^2^σ*/*τ) as the function of chemical potential (μ) at 300, 500 and 700 K are obtained, whereas the positive and negative μ correspond to n-type and p-type of monolayer MAs_2_. The electricity transport properties can be obtained by

(3) $$\sigma_{\alpha \beta }\left(T,\mu \right)=\frac{1}{V}\int \sum_{\alpha \beta }\left(\varepsilon \right)\left[-\frac{\partial f_{\mu }\left(T,\varepsilon \right)}{\partial \varepsilon }\right]d\varepsilon ,$$

(4) $$S_{\alpha \beta }\left(T,\mu \right)=\frac{1}{eTV\sigma_{\alpha \beta }\left(T,\mu \right)}\int \sum_{\alpha \beta }\left(\varepsilon \right)\left(\varepsilon -\mu \right)\left[-\frac{\partial f_{\mu }\left(T,\varepsilon \right)}{\partial \varepsilon }\right]d\varepsilon ,$$

(5) $$\sum_{\alpha \beta }\left(\varepsilon \right)=\frac{e^{2}}{N_{0}}\sum_{i,k}\tau \upsilon_{\alpha }\left(i,k\right)\nu_{\beta }\left(i,k\right)\frac{\delta \left(\varepsilon -\varepsilon_{i,k}\right)}{d\varepsilon },$$

where *α* and *β* are cartesian indices, *V* is the volume of the primitive cell and $\sum_{\alpha \beta }\left(\varepsilon \right)$ is the transport distribution function. The S is inversely proportional to temperature, which is proven in Figure 3a–c. We can clearly find that the absolute value of S of monolayer MAs_2_ decreases with the increase in temperature. The p-type and n-type doping of monolayer PdAs_2_ surprisingly possess a very large absolute value of S up to 440 and 460 μV/K at 300 K, which is significant to improve power factor. Meanwhile, the absolute value of p-type S of monolayer NiAs_2_ and PtAs_2_ are also observed about 140 and 135 μV/K, respectively. The calculated large S of monolayer MAs_2_ can be benefited from the PDOS.

The electrical conductively (σ) is one of the important parameters for analyzing thermoelectric properties. As Figure 3d–f presents, the pretty high σ can be observed, which is very beneficial for optimizing PF and thus improving thermoelectric performance. In addition, we can find that the change in σ is independent of the change in temperature, which is different from the trend of *S*. Then, further decomposition suggests that the σ of monolayer PdAs_2_ is less than that of monolayer NiAs_2_ and PtAs_2_, while the σ of n-type doping is always superior to that of p-type doping.

The electronic thermal conductivity (κ*_e_*) can be calculated by the Wiedemann-Franz law:

κ_e_ = LσT (6)

where L = π^2^κ_B_^2^/3e^2^ is the Lorenz number. From Figure 3g–i, we can clearly find that the function curve of *κ_e_* is similar to that of the *σ*, which is contributed by the proportional relationship between them.

According to Equation (1), the PF can be evaluated and illustrated in Figure 3j–l, which is obtained by combining *S* with *σ*. The maximum value of PF is n-type monolayer PdAs_2_ up to 3.9 × 10^11^ W/K^2^ms, which is much higher than n-type monolayer NiAs_2_ and p-type monolayer PtAs_2_ corresponding to 2.3 × 10^11^ W/K^2^ms and 1.7 × 10^11^ W/K^2^ms, respectively. This phenomenon is mainly caused by the dominant advantage of *S*. The calculated results suggest that monolayer MAs_2_ possess the great merits to be a promising thermoelectric material.

### 3.3. Thermal Transport Properties

**Figure 4:**
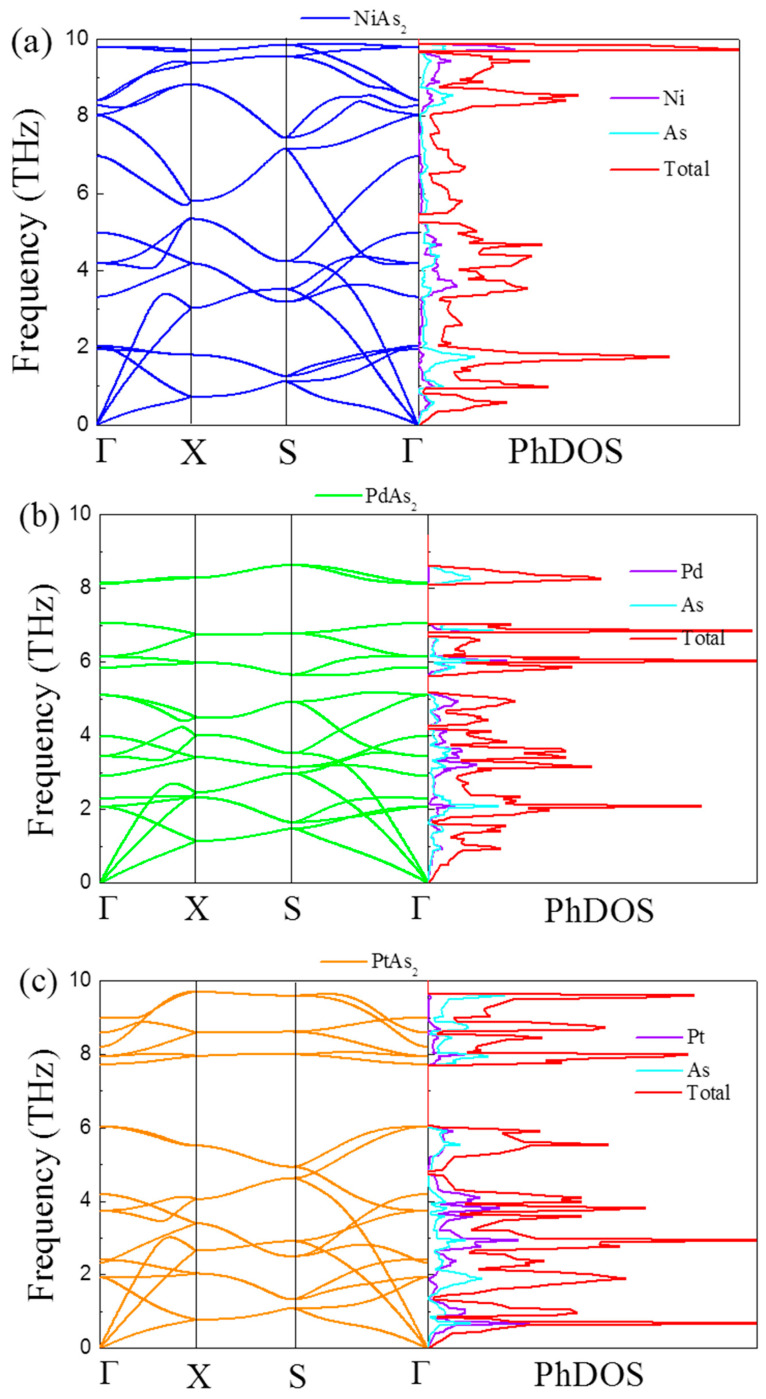
Phonon dispersion and corresponding phonon density of states (PhDOS) of monolayer (**a**) NiAs_2_, (**b**) PdAs_2_ and (**c**) PtAs_2_ along several high symmetry directions.

In order to accurately analyze the effect of phonon transport properties on TE performance, we computed the phonon spectrum of monolayer MAs_2_ and corresponding phonon DOS (PhDOS) as plotted in Figure 4. The lack of virtual frequency in the phonon spectrum indicates that monolayer MAs_2_ is dynamically stable, which is consistent with the previous theoretical date. There are two M and four As atoms, corresponding to eighteen curves which include three phonon–phonon and fifteen optical–phonon curves. Additionally, the phonon spectrum consisting of two parts that correspond to the two parts of PhDOS can be clearly observed. Among them, the phenomenon is that the low-frequency phonon–phonon curves are mainly controlled by the vibration of M atoms, while the vibrations of M and As atoms jointly contribute to the optical-phonon curves.

**Figure 5:**
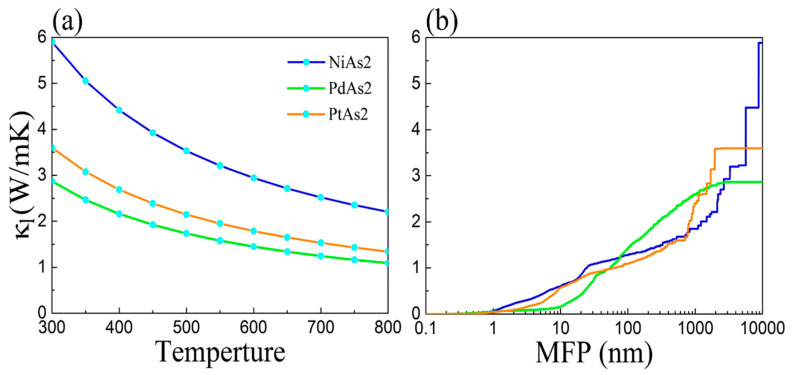
(**a**) Lattice thermal conductivities (*κ_l_*) of monolayer MAs_2_ as a function of temperature, and (**b**) the phonon mean free path (MFP) as a function of accumulated *κ_l_* of monolater MAs_2_ at 300 K.

The lattice thermal conductivity (*κ_l_*) is one of important factors for evaluating TE properties, which can be proved from Equation (2). Based on the Boltzmann transport theory with implementing in ShengBTE mode, the *κ_l_* of monolayer NiAs_2_, PdAs_2_ and PtAs_2_ can be computed by

(7) $$\kappa_{l}=\frac{1}{V}\underset{\lambda }{\sum }C_{\lambda }\nu_{\lambda }^{2}\tau_{\lambda }$$

where *C_λ_*, *v_λ_* and *τ_λ_* are the mode heat capacity, phonon group velocity and relaxation time, respectively. The *κ_l_* as a function of temperature is presented in Figure 5a. We can find that the *κ_l_* of monolayer MAs_2_ gradually reduce with the increase in temperature following the inverse relation, which is mainly caused by increasing phonon scattering with the elevating temperature. At 300 K, The *κ_l_* of monolayer NiAs_2_, PdAs_2_ and PtAs_2_ are 5.9, 2.9 and 3.6 W/mK, respectively.

The notion that nanostructures can effectively reduce thermal conductivity and thus improve thermoelectric performance has been proved, because nanostructures can hinder phonon transport and reduce the lattice thermal conductivity while having little impact on the electronic thermal conductivity, which greatly reduces the interaction between transport parameters. Consequently, the influence of size effect on *κ_l_* is considered and calculated. As Figure 5b demonstrates, the phonon mean free path (MFP) as a function of accumulated *κ_l_* of monolayer MAs_2_ at 300 K exhibits that the value is optical within the range of 1 nm, due to the accumulated *κ_l_* having no change with transforming size. Surprisingly, a positive phenomenon we can observe is that the slope curves of monolayer PdAs_2_ is very small, which can actively promote the application in TE materials.

**Figure 6:**
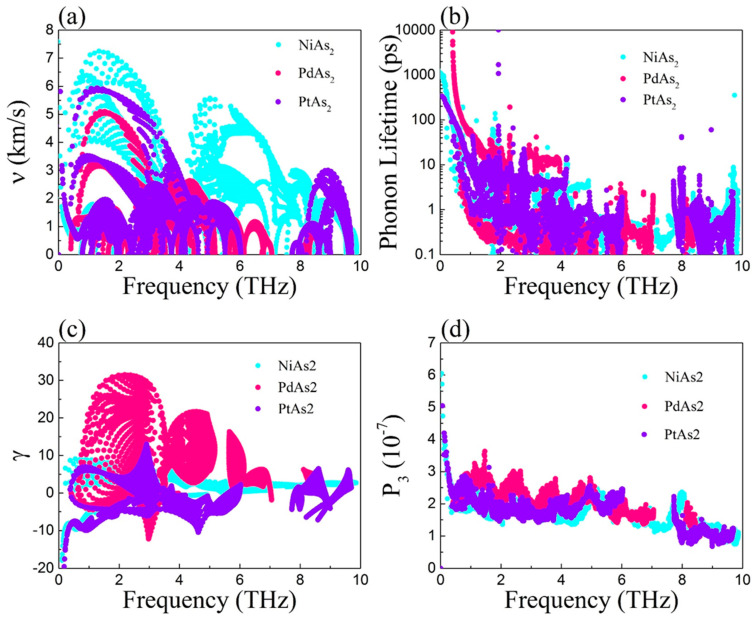
(**a**) Phonon group velocities, (**b**) phonon relaxation time, (**c**) Grüneisen parameters, and (**d**) P_3_ phase space with respect to frequency for MAs_2_.

In order to analyze the influence of lattice thermal conductivity on TE performance in detail, we calculated the phonon group velocity (*v*), relaxation time (*τ*), Grüneisen parameters (γ) and the three-phonon scattering phase space (P_3_) as presented in Figure 6.

An important factor (*v*) affecting the evaluation of thermal transport ability is determined by employing the phonon dispersion, which can be calculated by

(8) $$\nu_{\lambda q}=\frac{\partial \omega_{\lambda q}}{\partial q}$$

where ω*_λ,q_* is the phonon frequency. In Figure 6a, we can clearly find that the *v* of monolayer MAs_2_ of low-frequency acoustic breaches are much higher than that of high-frequency optical breaches, which indicates that acoustic breaches make a contribution to the *κ_l_*. The value of *v* in the low-frequency region at 300 K can be obtained of 7.6, 5.2 and 6 Km/s for NiAs_2_, PdAs_2_ and PtAs_2_, respectively. The order of magnitude is NiAs_2_ > PtAs_2_ > PdAs_2_, which suggests that the magnitude relationship of the *κ_l_* in Figure 5a is consistent. As Figure 6b shows, another key parameter phonon relaxation time (*τ*) is evaluated according to Equation (5). We can find that the *τ* of monolayer PtAs_2_ is smaller than that of monolayer NiAs_2_ and PdAs_2_, which is useful for receiving desired *κ_l_*.

Usually, the anharmonic interactions are used to determine the intensity of interactions and described by *γ*; thus, the greater anharmonic interaction can promote the generation of a much stronger phonon-phonon interaction as well as a smaller lattice thermal conductivity. Figure 6c displays the Grüneisen parameters (*γ*) of monolayer MAs_2_ with respect to frequency at 300 K, which can be calculated by

(9) $$\gamma \left(q\right)=-\frac{V}{\omega \left(q\right)}\;\frac{\partial \omega \left(q\right)}{\partial V}$$

where *V* is the volume. We can find that monolayer MAs_2_ possess a very high value for *γ* at a low frequency, corresponding to 10, 32 and 13 of NiAs_2_, PdAs_2_ and PtAs_2_, respectively. Obviously, the value of *γ* of monolayer PdAs_2_ is much higher than that of NiAs_2_ and PtAs_2_ sheet, indicating that monolayer PdAs_2_ has a large anharmonic interaction, which causes the smallest *κ_l_* in three arsenic compounds.

The three-phonon scattering phase space (P_3_) is used to describe the *τ*, and a larger value of P_3_ shows that more space is adopted to the three-phonon scattering, while a shorter *τ* can be reaped. As shown as Figure 6d, the P_3_ of monolayer MAs_2_ as function of phonon frequency is obtained. We can clearly find that monolayer MAs_2_ possess a large scattering phase space at a low frequency, indicating that they can promote little *τ* for acoustic phonon breaches.

### 3.4. Thermoelectric Figure of Merit (ZT)

**Figure 7:**
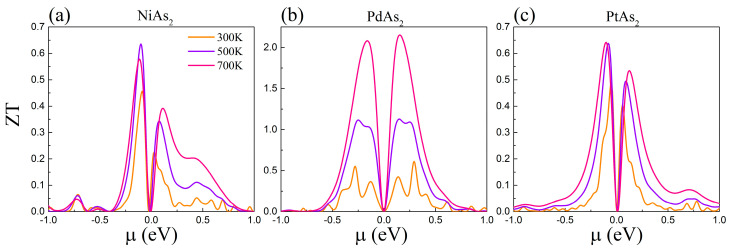
Thermoelectric figure of merit (ZT) value of monolayer (**a**) NiAs_2_, (**b**) PdAs_2_ and (**c**) PtAs_2_ as a function of chemical potential at 300, 500 and 700 K.

The large power factor (PF) and very low thermal conductivity of monolayer MAs_2_ are obtained through the calculation of electronic and phonon transport properties, i.e., a high thermoelectric figure of merit (ZT) is generated. By combining the phonon and electron transport coefficients, we calculate the ZT of monolayer MAs_2_.The electronic scattering time τ is obtained by the DP theory, as shown in Table 2. The ZT values of monolayer NiAs_2_, PdAs_2_ and PtAs_2_ as functions of chemical potential at 300, 500 and 700 K are plotted in Figure 7 corresponding to (a), (b) and (c), respectively. We can clearly note that the p-type doping ZT value of NiAs_2_ and PtAs_2_ sheet are greater than n-type doping, while the ZT value of sing-layer PdAs_2_ is contrary to them and belongs to n-type doping, which is consistent with the type of PF. The maximum ZT value of monolayer NiAs_2_ (p-type), PdAs_2_ (n-type) and PtAs_2_ (p-type) are 0.58, 2.1 and 0.64 at 700 K, respectively. The predicted *ZT* value of PdAs_2_ is larger than those of the commercial TE material p-type penta-PdX_2_ (X = S, Se) [41] and some other arsenic compound [42].

Besides, the ZT value of PdAs_2_ was three to four times higher than that of the other two arsenic compounds, mainly due to the combination of larger Seebeck coefficient and lower lattice thermal conductivity. The monolayer PdAs_2_ can be expected for application in thermoelectric material.

## 4. Conclusions

In summary, based on the first-principles calculations and combined with the Boltzmann transport theory, the TE properties of sing-layer NiAs_2_, PdAs_2_ and PtAs_2_ are confirmed. We verified that the crystal structure of monolayer MAs_2_ is dynamically stable. Subsequently, the phonon spectrum was analyzed in detail, including the lattice thermal conductivity, phonon group velocity, relaxation time, Grüneisen parameters, the three-phonon scattering phase space and the size effect. The calculated results indicate that the small group speed of 5.2 km/s and large Grüneisen parameters of 32 for PdAs_2_ promote the generation of a minimum lattice thermal conductivity of about 2.9 W/mK in three arsenic compounds. In addition, the calculated electron transport properties show that the Seebeck coefficients at 300 K of NiAs_2_ (p-type) and PtAs_2_ (p-type) are 140 and 135 μV/K, while n-type doping PdAs_2_ is 460 μV/K, which determines the types of power factor. According to Formula 1 and the combination of these data with the calculated results, the ZT values of monolayer NiAs_2_, PdAs_2_ and PtAs_2_ were calculated of the values 0.58 (p-type), 2.1 (n-type) and 0.64 (p-type) at 700 K, respectively, which suggests that MAs_2_ might become a promising thermoelectric material.

## Figures and Tables

**Table 1:** Lattice constants (LC) of 2D MAs_2_ (M = Ni, Pd, Pt) sheets and the degrees of α, β and γ angles.

	LC (Å)	α (deg)	β (deg)	γ (deg)
NiAs_2_	5.89	132.64	90	113.68
PdAs_2_	6.19	129.39	90	115.30
PtAs_2_	6.16	129.78	90	115.11

**Table 2:** Deformation potential (DP) constant E_l_, 2D elastic constants C^2D^, effective mass m*, carrier mobility μ, and scattering time τ for electron (e) and hole (h) along conveyor directions in the 2D monolayer MAs_2_ sheet at 300 K.

	Carriers Type	E_l_ (eV)	C^2D^ (J/m^2^)	m*/m_0_	μ (10^4^ cm^2^/Vs)	τ (ps)
NiAs_2_	electorn	2.2	100	0.14	1.47	1.17
	hole	1.68	100	0.16	1.93	1.76
PdAs_2_	electorn	1.52	93	0.21	1.27	1.51
	hole	0.69	93	0.09	34.27	1.75
PtAs_2_	electorn	1.41	117	0.07	17.07	6.79
	hole	2.33	117	0.08	4.80	2.18

## References

[1] Disalvo F.J. (1999). Thermoelectric cooling and power generation. Science.

[2] He J., Tritt T. (2017). Advances in thermoelectric materials research: Looking back and moving forward. Science.

[3] Zhu X., Liu P., Zhang J., Zhang P., Zhou W.-X., Xie G., Wang B. (2019). Monolayer SnP_3_: An excellent p-type thermoelectric material. Nanoscale.

[4] Zhang C., de la Mata M., Li Z., Belarre F.J., Arbiol J., Khor K.A., Poletti D., Zhu B., Yan Q., Xiong Q. (2016). Enhanced thermoelectric performance of solution-derived bismuth telluride based nanocomposites via liquid-phase Sintering. Nano Energy.

[5] Tang L.P., Tang L.M., Geng H., Yi Y.P., Wei Z., Chen K.Q., Deng H.X. (2018). Tuning transport performance in two-dimensional metal-organic framework semiconductors: Role of the metal d band. Appl. Phys. Lett..

[6] Pei Y., Wang H., Snyder G. (2012). Band Engineering of Thermoelectric Materials. Adv. Mater..

[7] Dun C., Hewitt C.A., Li Q., Guo Y., Jiang Q., Xu J., Marcus G., Schall D.C., Carroll D.L. (2017). Self-Assembled Heterostructures: Selective Growth of Metallic Nanoparticles on V2–VI3 Nanoplates. Adv. Mater..

[8] Zeng Y.-J., Wu D., Cao X.-H., Zhou W.-X., Tang L.-M., Chen K.-Q. (2020). Nanoscale Organic Thermoelectric Materials: Measurement, Theoretical Models, and Optimization Strategies. Adv. Funct. Mater..

[9] Wu Y.Y., Bo T., Zhu X., Wang Z., Wu J., Li Y., Wang B.T. (2020). Two-dimensional tetragonal Ti_2_BN: A novel potential anode material for Li-ion batteries. Appl. Surf. Sci..

[10] Wu Y.Y., Bo T., Zhang J., Lu Z., Wang Z., Li Y., Wang B.T. (2019). Novel two-dimensional tetragonal vanadium carbides and nitrides as promising materials for Li-ion batteries. Phys. Chem. Chem. Phys..

[11] Liu H., Neal A.T., Zhu Z., Luo Z., Xu X., Tománek D., Ye P.D. (2014). Phosphorene: An unexplored 2D semiconductor with a high hole mobility. ACS Nano.

[12] Medrano Sandonas L., Teich D., Gutierrez R., Lorenz T., Pecchia A., Seifert G., Cuniberti G. (2016). Anisotropic Thermoelectric Response in Two-Dimensional Puckered Structures. J. Phys. Chem..

[13] Sun X., Cronin S.B., Liu J., Wang K.L., Koga T., Dresselhaus M.S., Chen G. Experimental study of the effect of the quantum well structures on the thermoelectric figure of merit in Si/Si_1-x_Ge_x_ system. Proceedings of the 18th International Conference on Thermoelectrics.

[14] Hicks L.D., Harman T.C., Dresselhaus M.S. (1993). Use of quantum-well superlattices to obtain a high figure of merit from nonconventional thermoelectric materials. Appl. Phys. Lett..

[15] Sun X., Dresselhaus M.S., Wang K.L., Tanner M.O. (1997). Effect of quantum-well structures on the thermoelectric figure of merit in the Si/Si_1−x_Ge_x_ system. MRS Online Proc. Libr. Arch..

[16] Li Z.W. (2017). Thermoelectric properties of carbon nanotube/silicone rubber composites. J. Exp. Nanosci..

[17] Imae I., Kataoka H., Harima Y. (2019). Flexible thermoelectric materials based on conducting polymers doped with silicone polymer electrolyte. Mol. Cryst. Liq. Cryst..

[18] Kou L., Chen C., Smith S.C. (2015). Phosphorene: Fabrication, Properties, and Applications. J. Phys. Chem. Lett..

[19] Zhang J., Liu H.J., Cheng L., Wei J., Liang J.H., Fan D.D., Shi J., Tang X.F., Zhang Q.J. (2014). Phosphorene nanoribbon as a promising candidate for thermoelectric applications. Sci. Rep..

[20] Xu Y., Zhang H., Shao H., Ni G., Li J., Lu H., Zhang R., Peng B., Zhu Y., Zhu H. (2017). First-principles study on the electronic, optical, and transport properties of monolayer α- and β-GeSe. Phys. Rev..

[21] Jeong G., Jaung Y.H., Kim J., Song J.Y., Shin B. (2018). Sn1-xSe thin films with low thermal conductivity: Role of stoichiometric deviation in thermal transport. J. Mater. Chem..

[22] Zhang S., Zhou J., Wang Q., Chen X., Kawazoe Y., Jena P. (2015). Penta-graphene: A new carbon allotrope. Proc. Natl. Acad. Sci. USA.

[23] Shao Y., Shao M., Kawazoe Y., Shi X., Pan H. (2018). Exploring new two-dimensional monolayers: Pentagonal transition metal borides/carbides (penta-TMB/Cs). J. Mater. Chem..

[24] Liu H., Qin G., Lin Y., Hu M. (2016). Disparate strain dependent thermal conductivity of two-dimensional penta-structures. Nano Lett..

[25] Shojaei F., Hahn J.R., Kang H.S. (2017). Electronic structure and photocatalytic band offset of few-layer GeP_2_. J. Mater. Chem..

[26] Qian S., Sheng X., Xu X., Wu Y., Lu N., Qin Z., Feng E., Huang W., Zhou Y., Zhang C. (2019). Penta-MX_2_(M = Ni, Pd and Pt, X = P and As) monolayers: Direct band-gap semiconductors with high carrier mobility. J. Mater. Chem..

[27] Kresse G., Marsman M., Furthmüller J. (2014). Vienna Ab initio Simulation Package (VASP), The Guide. Computational Materials Physics.

[28] Blochl P.E. (1994). Projector augmented-wave method. Phys. Rev. Condens Matter.

[29] Perdew J.P., Zunger A. (1981). Self-interaction correction to density-functional approximations for many-electron systems. Phys. Rev..

[30] Perdew J.P., Burke K., Ernzerhof M. (1996). Generalized gradient approximation made simple. Phys. Rev. Lett..

[31] Heyd J., Scuseria G.E., Ernzerhof M. (2003). Hybrid functionals based on a screened Coulomb potential. J. Chem. Phys..

[32] Madsen G.K.H., Singh D.J. (2006). BoltzTraP. A code for calculating band-structure dependent quantities. Comput. Phys. Commun..

[33] Li W., Carrete J., Katcho N.A., Mingo N. (2014). ShengBTE: A solver of the Boltzmann transport equation for phonons. Comput. Phys. Commun..

[34] Togo A., Tanaka I. (2015). First principles phonon calculations in materials science. Scr. Mater..

[35] Zhang L.-C., Qin G., Fang W.-Z., Cui H.-J., Zheng Q.-R., Yan Q.-B., Su G. (2016). Tinselenidene: A Two-dimensional Auxetic Material with Ultralow Lattice Thermal Conductivity and Ultrahigh Hole Mobility. Sci. Rep..

[36] Bardeen J., Shockley W. (1950). Deformation Potentials and Mobilities in Non-Polar Crystals. Phys. Rev..

[37] Chaput L., Pécheur P., Scherrer H. (2007). Thermopower, Hall tensor, and relaxation time approximation for elemental zinc. Phys. Rev..

[38] Yuan H., Li Z., Yang J. (2018). Atomically thin semiconducting penta-PdP_2_ and PdAs_2_ with ultrahigh carrier mobility. J. Mater. Chem..

[39] Pan X.L., Zhao Y.Q., Zeng Z.Y., Chen X.R., Chen Q.F. (2020). Electronic, elastic, optical and thermal transport properties of penta-PdAs_2_ monolayer: First-principles study. Solid State Commun..

[40] Cai Y., Zhang G., Zhang Y.W. (2014). Polarity-Reversed Robust Carrier Mobility in Monolayer MoS_2_ Nanoribbons. J. Am. Chem. Soc..

[41] Lan Y.-S., Chen X.-R., Hu C.-E., Cheng Y., Chen Q.-F. (2019). Penta-PdX_2_ (X = S, Se, Te) monolayers: Promising anisotropic thermoelectric materials. J. Mater. Chem..

[42] Zhou Y., Zhao Y.-Q., Zeng Z.-Y., Chen X.-R., Geng H.-Y. (2019). Anisotropic thermoelectric properties of Weyl semimetal NbX (X = P and As): A potential thermoelectric material. Phys. Chem. Chem. Phys..

